# SysPTM 2.0: an updated systematic resource for post-translational modification

**DOI:** 10.1093/database/bau025

**Published:** 2014-04-03

**Authors:** Jing Li, Jia Jia, Hong Li, Jian Yu, Han Sun, Ying He, Daqing Lv, Xiaojuan Yang, Michael O. Glocker, Liangxiao Ma, Jiabei Yang, Ling Li, Wei Li, Guoqing Zhang, Qian Liu, Yixue Li, Lu Xie

**Affiliations:** ^1^Key Laboratory of Biomedical Photonics of Ministry of Education, College of Life Science and Technology, Huazhong University of Science and Technology, Wuhan 430074, P. R. China, ^2^Shanghai Center for Bioinformation Technology, Shanghai Institutes of Biomedicine, Shanghai Academy of Science and Technology, Shanghai 201203, P. R. China, ^3^Britton Chance Center for Biomedical Photonics, Wuhan National Laboratory for Optoelectronics, Huazhong University of Science and Technology, Wuhan 430074, P. R. China, ^4^Department of Bioinformatics and Biostatistics, Shanghai Jiaotong University, Shanghai 200240, P. R. China, ^5^Key Laboratory of Systems Biology, Chinese Academy of Sciences, Shanghai 200031, P. R. China and ^6^Proteome Center Rostock, Department for Proteome Research, Institute of Immunology, University of Rostock, Rostock 18055, Germany

## Abstract

Post-translational modifications (PTMs) of proteins play essential roles in almost all cellular processes, and are closely related to physiological activity and disease development of living organisms. The development of tandem mass spectrometry (MS/MS) has resulted in a rapid increase of PTMs identified on proteins from different species. The collection and systematic ordering of PTM data should provide invaluable information for understanding cellular processes and signaling pathways regulated by PTMs. For this original purpose we developed SysPTM, a systematic resource installed with comprehensive PTM data and a suite of web tools for annotation of PTMs in 2009. Four years later, there has been a significant advance with the generation of PTM data and, consequently, more sophisticated analysis requirements have to be met. Here we submit an updated version of SysPTM 2.0 (http://lifecenter.sgst.cn/SysPTM/), with almost doubled data content, enhanced web-based analysis tools of PTMBlast, PTMPathway, PTMPhylog, PTMCluster. Moreover, a new session SysPTM-H is constructed to graphically represent the combinatorial histone PTMs and dynamic regulation of histone modifying enzymes, and a new tool PTMGO is added for functional annotation and enrichment analysis. SysPTM 2.0 not only facilitates resourceful annotation of PTM sites but allows systematic investigation of PTM functions by the user.
**Citation details:** Li,J., Jia,J., Li,H. *et al.* SysPTM 2.0: an updated systematic resource for post-translational modification. *Database* (2014) Vol. 2014: article ID bau025; doi:10.1093/database/bau025.

**Citation details:** Li,J., Jia,J., Li,H. *et al.* SysPTM 2.0: an updated systematic resource for post-translational modification. *Database* (2014) Vol. 2014: article ID bau025; doi:10.1093/database/bau025.

**Database URL:** http://lifecenter.sgst.cn/SysPTM/

## Introduction

Protein post-translational modifications (PTMs) regulate physicochemical properties, maturity and activity of most proteins, and play crucial roles in many cellular processes. For example, reversible phosphorylation is implicated in cell cycle, cell growth, apoptosis and signal transduction (1, 2); methylation at certain residues of histones can activate or repress gene expression (3); and SUMOylation of transcriptional regulators results in the inhibition of gene transcription (4). The development of mass spectrometry alongside improved protein separation and enrichment technology (5, 6) resulted in more and more studies on proteome-wide PTM substrates, and the rate of identification of PTM sites is considerably outpacing our biological knowledge of the function of these modifications (7). Such progress further fuels the construction of various PTMs repositories, which proved to be invaluable sources for understanding the function of PTMs.

Currently, most PTM repositories mainly focus on a specific modification type. O-GLYCBASE (8) focuses on glycoproteins and their O-linked glycosylation sites. Phospho.ELM (9) and Phosphorylation Site Database (10) are the databases of phosphorylation sites, and PHOSIDA (11) store mainly serine-, threonine-, and/or tyrosine-phosphorylated proteins and phosphorylation site information. PTM site information for a particular protein can also be found in protein reference databases like UniProt Knowledgebase (12) and HPRD (13), but the main purpose of these databases is to provide comprehensive annotations for all proteins. Compared to single type-annotation or scattered multi-type annotations of proteins carrying PTMs, integrated PTM databases are being developed as well, to provide a more global view of PTMs. For example, dbPTM 3.0 (14) integrates both the experimentally validated and computationally predicted PTM sites of proteins from various resources. It also provides the substrate specificity of PTM sites and functional association between PTM substrates and their interacting proteins. PhosphoSitePlus (15) provides comprehensive information and tools for the study of phosphorylation, ubiquitination, acetylation and methylation. Another newly published database, PTMcode (16) integrates 13 commonly studied PTM types across eukaryotes and displays the potential co-regulations and functional associations of collected PTMs deduced from the co-evolution analysis of modified residues.

With emphases on curating modification data from large-scale tandem mass spectrometry (MS/MS) experiments and providing in-depth online analysis engines for PTM proteins, our work SysPTM (17) was developed as a comprehensive resource integrated with existing features of numerous external databases, curated MS/MS data and four analysis tools (PTMBlast, PTMPathway, PTMPhylog, PTMCluster). The first version of SysPTM was released in 2009 and has been well used since. For instance, SysPTM datasets were used to develop computational models for prediction of protein *S*-nitrosylation sites (18) and protein lysine acetylation sites (19). Li *et al.* (20) performed a comprehensive annotation of phosphoproteome of mouse embryonic stem cells by using SysPTM datasets and tools. Schweiger and Linial (21) discovered the cooperativity within proximal phosphorylation sites by using information derived from SysPTM.

Four years after we constructed the database, there have been significant advances over the generation of various types of PTM data. The new version of the SysPTM 2.0 we release now results in more than doubled data content, i.e. 471 109 PTM sites on 53 235 proteins, covering over 50 modification types across 2031 species, detailed with widened functional annotation derived from MS/MS experiments and various public data resources. The utilities of four analysis tools (PTMBlast, PTMPathway, PTMPhylog, PTMCluster) have been greatly improved to support batch query and online calculation analysis processes of relevant biological functions of PTMs. In addition, a new session, SysPTM-H, is developed to graphically represent the combinatorial histone PTMs and dynamic regulations of histone modifying enzymes. A fifth tool, PTMGO, is implemented to facilitate a better understanding of PTM events in complex biological processes.

## Data Sources

**Figure 1.**
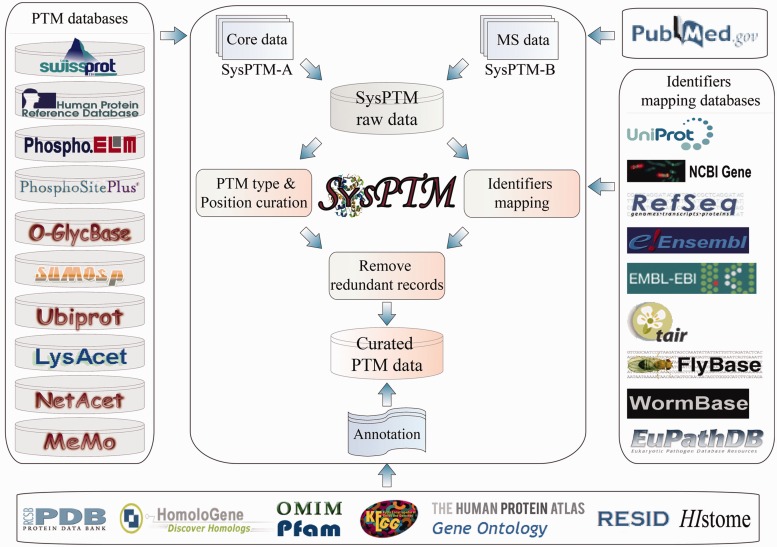
PTM data sources and process procedures employed by SysPTM2.0.

As in the previous version, PTM data in SysPTM 2.0 are integrated into two datasets, SysPTM-A and SysPTM-B, with PTM sites collected from public data resources and peer reviewed MS/MS literature, respectively. Concerted histone modifications were not specifically notified in the previous SysPTM version. But they are of such important functional consequence and research interest, that we added a new session SysPTM-H this time, with curated PTM sites from five major types of histone proteins (H1/H5, H2A, H2B, H3 and H4) (22). Data were processed as demonstrated in Figure 1: (i) SysPTM-A integrated PTM sites and substrates from 10 external resources: version 6.0 of O-GLYCBASE (8), version 9.0 of Phospho.ELM (9), version 1.0 of PhosphoSitePlus (15), UniProtKB/Swiss-Prot (release 2012_05) (12), release 9 of HPRD (13), version 1.0 of UbiProt (23), version 1.0 of SUMOsp (24), version 2.0 of Memo (25), version 1.0 of NetAcet (26) and version 1.1 of LysAcet (27). A Perl program was developed to retrieve and integrate PTM data automatically from these databases. (ii) SysPTM-B included literature-reported proteomic PTMs after MS/MS quality control and PTM scoring. Combinations of seven modification types (phosphorylation, acetylation, methylation, SUMOylation, ubiquitination, glycosylation, S-nitrosylation) and MS-related keywords (mass spectrometry, proteomics) were used to search PubMed (28) for the period of October 2008 to April 2013. Approximately 2420 research and review papers associated with MS/MS proteomics and protein modifications were retrieved. Only 299 qualified papers were selected, after manual check of the MS/MS data. (iii) To control the data quality, PTM data in SysPTM-A and SysPTM-B went through a rigorous screening process as described in our previous work (17). Because it is unfeasible to set standard score thresholds for PTM sites from different datasets with diverse experimental procedures, each dataset was controlled according to the data qualification in the corresponding original paper. In brief, only papers with intact PTM datasets and detailed PTM identification procedures were selected, and the datasets in these papers were used only if at least one of the following conditions was satisfied: (a) All spectra of modified peptides were manually validated; (b) Modified peptides were filtered by software score thresholds or false discovery rate (FDR); (c) Modified peptides were validated by proper PTM site localization algorithms (e.g. Ascore). Moreover, identifiers or names of PTM proteins extracted from MS/MS papers or external resources were mapped to protein UniProtKB accession numbers by using the *ID Mapping Service* at UniProt (13). The full-length protein sequences at UniProtKB were used as references to validate the correctness of identified PTM sites. Residues that could not align exactly to the corresponding protein sequence were discarded. (iv) SysPTM-H included histone PTM sites from original SysPTM-A and SysPTM-B, Histome (29) and relevant review papers (30, 31). The protein and gene expression of each individual modifying enzyme and demodifying enzyme of histone were collected from the Human Protein Atlas (32). (v) Information derived from KEGG (33), GO (34) and Pfam (35) were used to improve the annotation of PTM proteins in addition to the features provided by UniProtKB/Swiss-Prot (13). All PTM types were also cross-linked to the physiochemical properties stored in dbPTM 3.0 (15). In our database we also integrated, or linked to, annotation information from the following sources: PDB (36), OMIM (37), Ensembl (38), RefSeq (28), TAIR (39), FlyBase (40), WormBase (41), EuPathDB (42) and RESID (43).

## Improvement of Database Contents

**Figure 2.**
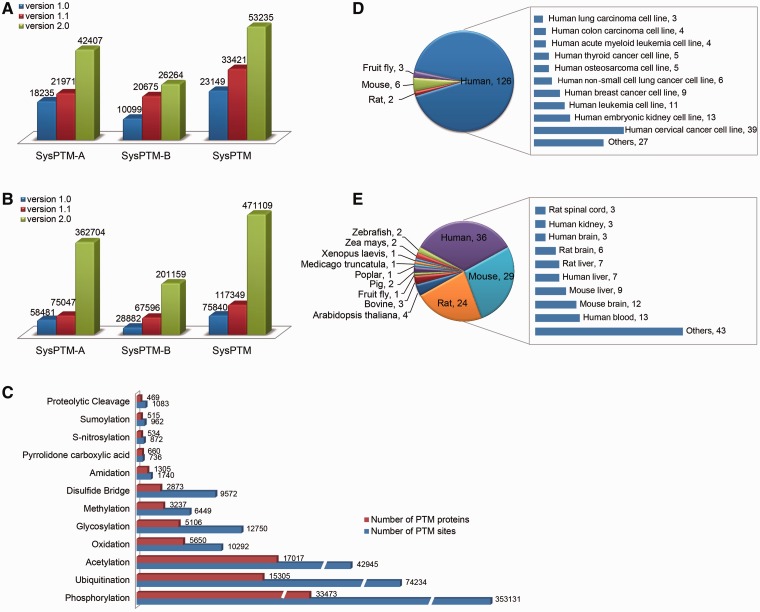
Data content in SysPTM2.0 and comparison to the previous database. (**A**) The growth number of unique PTM proteins in SysPTM-A, SysPTM-B and total database; (**B**) The growth number of unique PTM sites, in SysPTM-A, SysPTM-B and total database; (**C**) Number of experimentally validated PTM proteins and modified sites in 11 highly frequent modification types; (**D**) Number of cell-lines and their derived species stored in SysPTM-B; (**E**) Number of tissues and their derived species stored in SysPTM-B.

SysPTM-A contains 42 407 unique proteins and 362 704 modification sites collected from publicly available resources. SysPTM-B contains 26 264 unique proteins and 201 159 modification sites collected from 299 MS/MS papers. In total, the current version of SysPTM houses information of 471 109 PTM sites on 53 235 proteins, covering more than 50 modification types across 2031 species. Supplementary Figure S1 displays 20 species with the most abundant PTM data, including human, mouse, fruit fly, rat, C. elegans, Baker's yeast. Comparing to the previous version, SysPTM 2.0 is almost doubled in data content of unique PTM proteins (Figure 2A), and accordingly there is a 4-fold increase for unique PTM sites (Figure 2B). The distribution pattern of PTM proteins and PTM sites is shown in Figure 2C. Protein phosphorylation is still the PTM most frequently identified by experiments, whereas ubiquitination is the fastest-growing modification type studied during the past 4 years (Supplementary Table S1). Other important modifications include oxidation, acetylation and glycosylation.

Protein PTMs are important in many different biological processes, and their consequential functions can differ widely. Parallel comparison of PTMs occurring in complex biological processes is useful in identifying the differential regulation of PTMs. We therefore categorized 47 677 modified proteins into 287 KEGG reference pathways and 38 708 GO terms across 6 species: human, mouse, rat, fruit fly, zebrafish and Baker's yeast (The procedures are shown in Supplementary Methods). In addition, we also provide active links to access analysis of these subsets of data.

It is also known that the distribution of PTM types and modification sites varies under different biological conditions. Since data in SysPTM-B were collected with detailed sample information mined from MS/MS experiments, we further compartmentalized the PTM proteins and their sites into cell-lines or tissues from where they originate. In total, we mined 72 types of cell-lines from 141 MS/MS papers, and 79 tissues from 106 MS/MS papers. The statistics of cell-lines and tissues used in PTM studies are depicted in Figure 2D and E. Sixty-six human cell-lines were commonly used in global studies of PTM and 83.3% of these were cancer-derived human cells. The remaining six cell-lines belong to mouse, fruit fly, rat and monkey (Figure 2D). Supplementary Table S5 lists the experimentally verified substrate and modification sites in each biological cell-line. Various tissues derived from human, mouse and rat were used to study PTM profiles on proteome (Figure 2E). Human blood, human liver, mouse brain and mouse liver are the most prevalent samples used (Supplementary Table S6).

The session of SysPTM-H is another important PTM subset with the purpose of interpreting the dynamic regulation of histone PTMs by integrating expression profiles of histone modifying enzymes. It contains 1673 PTM sites on 288 unique histone proteins (Table 1). We collected 101 histone modifying enzymes (e.g. histone acetyltransferases, histone methyltransferases, ubiquitinases, etc.) and 52 demodifying enzymes (e.g. histone deacetylases, histone demethylases, deubiquitinases, etc.) (Supplementary Table S3). The protein and mRNA expression levels of these enzymes were collected from nine human cancer cell-lines that are commonly used in proteome-wide MS/MS experiments, such as MCF-7 from breast cancer, and A-431 from skin cancer (Supplementary Table S4). Thus, we are able to explore the potential co-regulation patterns of histones by comparing the expression variation of their modifying enzymes under different disease conditions.

**Table 1.** The statistics of unique histone PTMs and modification sites in SysPTM-H

Histone family	Number of PTM proteins	Number of PTM sites
H1/H5	66	407
H2A	65	250
H2B	69	482
H3	52	379
H4	36	155

## New Features in SYSPTM 2.0

### Enhanced PTM analysis tools

**Figure 3.**
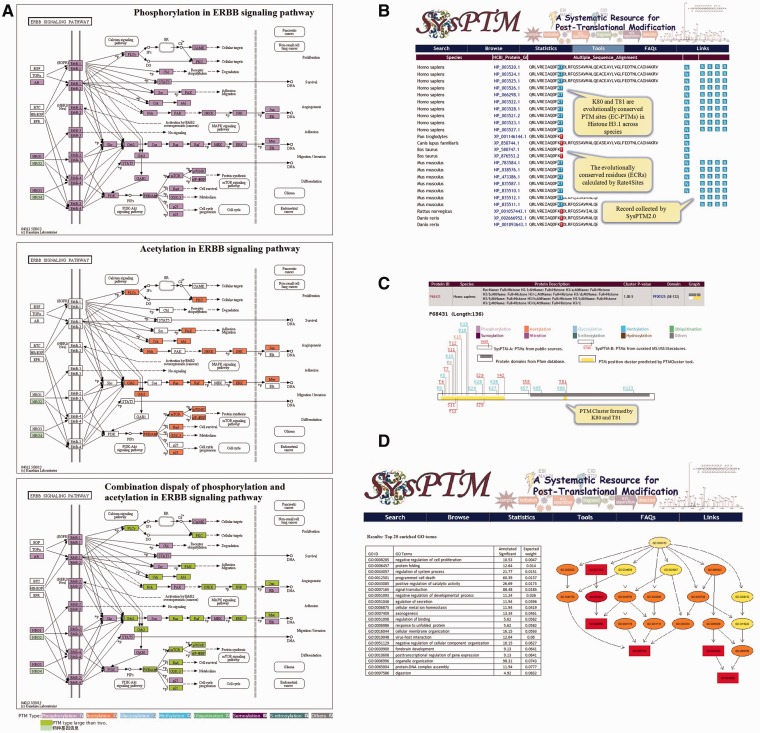
Analysis tools and their enhanced functions in SysPTM2.0. (**A**) Exploration of ERBB signaling pathway regulated by phosphorylation and acetylation in both individual and combinatorial manners. PTMs on pathways are colored by mapping user-queried proteins into the KEGG reference pathways. Each color indicates a specified PTM type, e.g. purple denotes phosphorylation, orange denotes acetylation, green box indicates the presence of multiple modifications in one protein; (**B**) PTMPhylog searching result of human H31 protein (P68431). ECRs calculated by Rate4Sites are represented with red background, and EC-PTMs are colored with blue background; (**C**) PTM cluster result of human H31 (P68431) calculated from PTMCluster. The known PTM site clusters can be queried by either keywords or protein sequences at PTMCluster. User can also upload or define PTM sites to calculate site clusters in a real-time manner. Protein domains are shown in gray and site clusters are shown by yellow. PTM sites contained in the cluster are marked in the upper and lower sides of the protein box (*upper*: PTM sites from SysPTM-A, *lower*: PTM sites from SysPTM-B); (**D**) The top 20 enriched GO terms identified by PTMGO using human proteome acetylation data in (57). The top enriched GO terms were identified by the elim algorithm. Rectangles indicate the most significant terms. Color represents the relative significance, ranging from dark red (most significant) to bright yellow (least significant). The GO identifier is displayed for each node.

Four online tools had been developed in SysPTM, including PTMBlast, to compare a user’s PTM dataset with PTM data in SysPTM; PTMPathway, to map PTM proteins to KEGG pathways; PTMPhylog, to discover potentially conserved PTM sites; and PTMCluster, to find clusters of multi-site modifications (17). These four tools had been proven useful by our case study and users of SysPTM in systematic PTM data analysis. Together with the update of SysPTM 2.0, the functions of the four existing PTM analysis tools have been updated and enhanced, and in addition a new tool named PTMGO was developed, to support a GO enrichment analysis of queried PTM proteins (highlighted in Figure 3).

#### PTMBlast

PTMBlast can be used to identify novel PTM sites by performing sequence alignment between user-defined PTM sites/peptides with different target datasets in SysPTM 2.0. Three sequence alignment methods were incorporated, and now displayed in three individual pages, namely PTMBlast, PTMBlast-SWA and PTMBlast-IWA. PTMBlast adopts the homology search against PTM sequences using the BLASTP program. PTMBlast-SWA employs Smith–Waterman algorithm (SWA) to identify known PTMs when queried by short peptides (with higher sensitivity) (44). PTMBlast-ISA incorporates an identical sequence alignment (ISA) method that requires protein sequences between query and subject must be identical, and is particularly useful for searching exactly identical PTM residues from MS/MS-derived peptides.

#### PTMPathway

Site-specific modification of proteins such as phosphorylation, ubiquitination and acetylation are involved in virtually all signaling pathways that orchestrate fundamental cellular processes, like cell cycle progression, apoptosis, DNA damage response, autophagy and metabolism (45). Pathway analysis using KEGG reference pathways could provide means to study how PTMs coordinate in cell signaling. PTMPathway in SysPTM 2.0 provides an upgraded interface and visualization solution to characterize the cell signaling modification status using KEGG API (33). One color is defined to represent a specific type of PTM, e.g. purple indicates phosphorylation and orange denotes acetylation, etc., and each PTM type can be optionally selected and displayed according to the user’s interest. Users can investigate two or more modification types of proteins by selecting one PTM type at one time, and then selecting a different PTM type, and so on. For nodes with different types of PTMs, different colors will show up on graph; as for a node with two or three PTMs occurring on the same site, the color will change to an even one (defined as both or all selected types of modifications are present). This function can help users clearly see how two or more different PTM types affect different proteins in the same pathway. Figure 3A shows exploration of the ERBB signaling pathway regulated by phosphorylation and acetylation in both individual and combinatorial manners, and in this way potential co-regulation of different PTM types in a signaling pathway cascade may also be revealed.

#### PTMPhylog

Highly conserved residues often play an essential role in the structure or function of proteins, and residue conservation for PTM types has been reported to demonstrate functional importance (46–49). In SysPTM 2.0 the evolutionally conserved residues (ECRs) of protein sequences influencing PTMs are identified by using ortholog groups from HomoloGene (28) and the Rate4Site algorithm (50). Rate4Site is an accurate and sensitive method for calculating the evolutionary rate at an amino-acid site to evaluate the residue conservation tendency (51). In SysPTM 2.0, the amino-acid sites with conservation scores higher than 0.9 are considered as ECRs (52, 53), and PTM sites occurring in a window of five residues to the ECRs are defined as ECRs-associated PTM sites (EC-PTMs) (The window size is the length of the average interval between two PTMs calculated from our data archives.). Figure 3B demonstrates the discovered ECRs and EC-PTMs at lysine 80 and threonine 81 of human H31 protein (P68431), highlighted by red and blue color, respectively, in the interface of PTMPhylog. In total, we detected 32 495 EC-PTMs from 357 890 ECRs. A further analysis suggests that 33.2% of EC-PTMs are located in the protein domains annotated by Pfam, whereas 54.2% conserved PTM sites preferably locate in ‘disordered regions’, i.e. less structured parts of proteins. This supports the finding that phosphosites are generally more conserved in the ‘disordered regions’ in vertebrate-specific functional modules (47) and is consistent with the assumptions that (i) ‘disordered regions’ are readily accessible by modifying enzymes; and (ii) a side-chain modification results in a structural (and consequently functional) change more rapidly with respect to altering solidly folded domains.

#### PTMCluster

It has previously been shown that some PTM sites and PTM types can form clusters that act as regulatory centers, such as the highly modified cassette of amino acids in p53 (54) and those extensively studied on histone H3/H4 N-terminal tails (31). To generalize such physical interactions to all PTM types and identify regions of PTM clusters, PTMCluster in SysPTM 2.0 is designed to perform non-parametric comparison of the distances between the modified residues by calculating the local peaks of PTMs with an improved approach on a neighborhood model proposed by Li *et al.* (55). Figure 3C shows that methylation on lysine 80 and phosphorylation on threonine 81 are a cluster on human H31 protein (P68431). A recent study reported that a methylation and phosphorylation dual modification on lysine 80 and threonine 81 located in the nucleosome core of H3 is primarily associated with mitotic chromosomes (56). The online calculation of PTM clusters was not available in the previous version. We now also provide the mapping between PTM clusters and the Pfam domains of proteins (Figure 3C). A total of 25 295 cluster peaks in 19 728 unique proteins are identified by PTMCluster. The largest cluster holds 190 PTM sties, and the most PTM abundant protein has 16 PTM clusters. PFAM domains cover 32.6% centers of identified PTM clusters.

#### PTMGO

It is known that PTM patterns may vary depending on cellular functions to be performed (57). Enrichment of over-represented GO terms from a list of interested proteins is an often used strategy in exploring functionally associated regulation mechanisms. PTMGO is added in SysPTM 2.0, to facilitate a better understanding of PTM events in complex biological processes. PTMGO is implemented through a gene enrichment analysis tool, topGo (topology-based Gene Ontology scoring) (58). PTMGO also supports comparison analysis of enriched GO terms between different biological samples. Figure 3D demonstrates a PTMGO analysis of rat and human lysine acetylation sites with phosphorylation sites, revealing organ specificity and subcellular patterns (57).

### Enhanced web interface

To facilitate the use of SysPTM 2.0 resource, the web interface has been redesigned. First, the search engine is enhanced by allowing batch request of PTM information using protein name, UniProtKB ID, or accession number, protein sequence, or modification site, with a maximum of 10 000 records. This provides a remarkable utility to perform more systematic and speedy proteome-wide PTM analyses.

**Figure 4.**
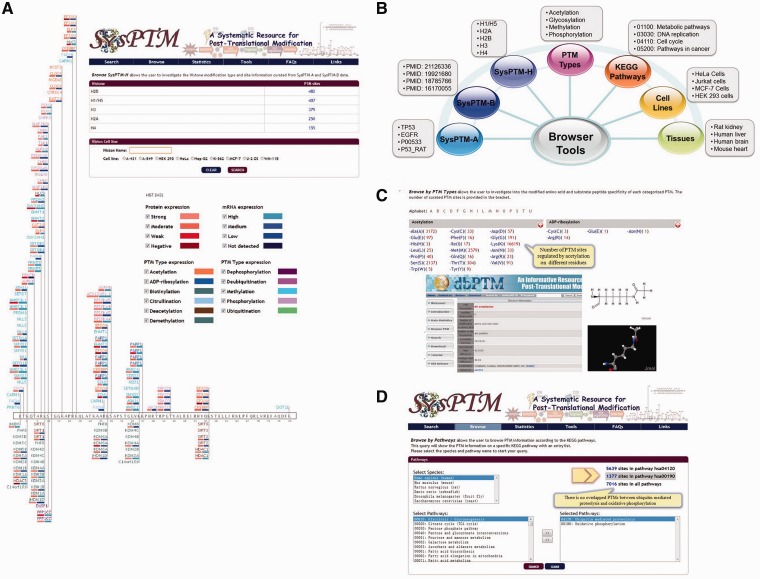
The web interfaces of SysPTM2.0 browser tools. (**A**) The page of enzyme-modified human histone H31 (P68431) in A-431 cell-line. PTM modifying and demodifying enzymes are separately displayed in the upper and lower sides of the protein box. Types of modification of enzymes are distinguished by text colors, e.g. purple denotes phosphorylation, and orange denotes acetylation, etc. Modifying and demodifying enzymes are also highlighted with red and blue background to represent the expression at both protein and mRNA level, respectively. A darker color represents a higher level of expression values. (**B**) Browser tools at SysPTM 2.0. (**C**) Browse by PTM types page. The overview of PTM types and their modified residues is provided, more detailed information of the catalytic specificity of PTM type can be obtained from dbPTM 3.0. (**D**) Enhanced function of PTMPathway. Users can explore multiple signaling pathways and compare PTM proteins and modified residues by searching SysPTM 2.0.

Second, in addition to general browsing of SysPTM-A or -B, SysPTM-H can now be browsed to display histone variants, their PTM sites and dynamic regulation of histone modifying enzymes. Disease-associated histone modification patterns can be observed by querying in combination a histone name and a cancer cell-line, as shown in Figure 4A. Differential expression of regulating enzymes may affect epigenetic reprogramming events in different samples (59). In addition to general browsing, it is also possible to retrieve PTM information from different perspectives, such as PTM type, KEGG pathway, GO term, biological sample, etc., as shown in Figure 4B. We also provide cross-linking to dbPTM 3.0 for detailed information of the catalytic specificity related to modified residues (Figure 4C). When browsing by cell-lines, tissues, KEGG pathways and GO terms, SysPTM 2.0 allows different entrances to quickly navigate PTMs involved in different physiological and biological processes. The full list of cell-lines and tissues are displayed in Supplementary Tables S5 and S6. In the interface of KEGG pathways and GO terms, it is also possible to explore multiple signaling pathways, molecular functions, biological processes or subcellular locations simultaneously, so that users may discover or visualize multi-functions of PTMs using SysPTM 2.0 (Figure 4D).

Third, the interface of PTM proteins is reframed in eight sections to represent the comprehensive annotation of each individual protein and their modification site information, namely protein information, PTMprotein-Annotation, PTMsite-Statistics, PTMsite/Peptide in sequence, PTMsite-Map, Protein/Peptide-Map, PTMsite-Table and PTMsite-Cluster. In the section of ‘protein information’, previously defined SysPTM ID is replaced by UniProtKB accession number for easier management of the data. This section also includes protein ID, protein name and synonyms, species, gene names and three-dimensional structure (Supplementary Figure S2A). ‘PTMprotein-Annotation’ displays the protein annotation from external public data sources, such as domains from Pfam. ‘PTMsite-Statistics’ shows the number of PTM sites for each modification type, along with the data source (Supplementary Figure S2B). ‘PTMsite/Peptide in sequence’ highlights the modification sites of different PTM types on the protein sequence. Seven most studied PTM types are highlighted by different colors in protein sequences, and green color indicates multi-modification events on a single residue (Supplementary Figure S2D). As a newly displayed part, ‘Protein/Peptide Map’ visualizes the PTMs and their associated conservation sites on genome datasets, with a graphical protein sequence viewer (Supplementary Figure S2F). By analysing the conservation of the original encoding genomic sequences of protein-modified substrates, a deeper understanding of PTMs can be taken from the genomic level. By comparing genomic conservation with conserved PTM sites predicted by PTMPhylog, biological evolution of PTM sites from genomic to proteomic level can be revealed. For the previously well-established tools of ‘PTMsite-Map’ (Supplementary Figure S2E), ‘PTMsite-Table’ and ‘PTMsite-Cluster’ (Supplementary Figure S2C), we retain their functions to display all information related to a protein PTM site such as data source, integrated annotation, predicted and calculated *P*-values, etc. either graphically or in tabular form.

## Conclusion and Future Directions

We have witnessed the beginning and significant acceleration of PTM identification by MS/MS. Research spotlights have encompassed PTM network analysis, PTM co-regulation and PTM site predictions, etc (16, 60–63). Fundamental to all is the construction of systematic databases to bear up such research projects. We believe SysPTM 2.0 to be one of such systematic resources, with comprehensive data resource and systemic online analysis tools to facilitate annotation of PTM sites and detailed investigation of PTM functions. However, we also see the potential needs of continuous updates and improvements that have to be carried on in the future. We expect an ever increasing number of data sources growing from various external databases and a large number of literature reports. For example, currently the histone modifying enzymes and their expression data are only derived from nine human cancer cell-lines, those from other human samples, and from model species such as mouse or rat await exploration. We expect web-based utilities in SysPTM to become more integrated with PTM functionality analysis. For example, a great number of PTMs detected by high throughput mass spectrometry are with ambiguous function, a scoring system incorporating information of PTMPhylog and PTMcluster should help to predict the functionalities of such PTMs.

## Supplementary Data

Supplementary Data are available at *Database* Online.
